# The Small Molecule SR8278 Inhibits Cell Proliferation Independent of the REV-ERB Nuclear Receptor Proteins in Human Keratinocytes

**DOI:** 10.3390/biom16030416

**Published:** 2026-03-12

**Authors:** Ushaswini Atluri, William Cvammen, Michael G. Kemp

**Affiliations:** 1 Department of Pharmacology and Toxicology, Wright State University Boonshoft School of Medicine, Dayton, OH 45435, USA; atluri.27@wright.edu (U.A.); 2 Department of Pharmacology and Chemical Biology, University of Pittsburgh, Pittsburgh, PA 15232, USA; whc26@pitt.edu (W.C.); 3 Dayton Veterans Affairs Medical Center, Dayton, OH 45428, USA

**Keywords:** cell proliferation, small molecule, circadian, REV-ERB, DNA synthesis, nuclear receptor, keratinocytes

## Abstract

The small molecule SR8278 was initially identified as an antagonist of the REV-ERB (reverse c-ERBAa) nuclear receptor proteins, which play important roles in metabolism and circadian rhythms. Though SR8278 has been shown to have beneficial physiological effects in a variety of different preclinical disease contexts, its impact on gene expression and cell proliferation in keratinocytes has not previously been examined. We therefore carried out an RNA-seq analysis and found that genes involved in the G1/S transition of the cell cycle were significantly impacted by SR8278 treatment, and these effects were confirmed at both the RNA and protein level by RT-qPCR and Western blotting, respectively. Cell proliferation assays showed that SR8278 slowed cell growth but did not induce genotoxic stress or apoptosis. Finally, the use of CRISPR/Cas9 genome editing and siRNA-mediated disruption of REV-ERB gene expression showed that the loss of the REV-ERB proteins did not impact the effect of SR8278 on gene expression and cell proliferation. We conclude that the anti-proliferative effects of SR8278 are not mediated by the REV-ERB proteins, and, thus, care should be taken when interpreting studies involving this compound unless complementary genetic approaches are also shown, particularly in studies involving cell proliferation.

## 1. Introduction

The *NR1D1* and *NR1D2* genes (nuclear receptor subfamily 1 group D members 1 and 2) encode the REV-ERB alpha (REV-ERBα) and REV-ERB beta (REV-ERBβ) nuclear receptor proteins, which utilize heme as a natural ligand [1,2] and compete with retinoic acid-related orphan receptors (RORs) for binding to ROR-response elements (ROREs) in the promoters of target genes [3]. The REV-ERBs have historically been thought to function as transcriptional repressors via the recruitment of the nuclear co-repressor (NCoR)/histone deacetylase 3 (HDAC2) complex. However, recent work indicates that in cancer cells, REV-ERBα becomes a transcriptional activator by interacting with BRD3/p300 to drive the expression of thousands of genes involved in tumorigenesis, including genes involved in MAPK and PI3K-Akt signaling [4].

Nonetheless, the major known functions of the REV-ERBs are in circadian rhythms and metabolism [5]. The REV-ERBs are both transcriptionally activated by the CLOCK (circadian locomotor output kaput)-BMAL1 (brain and muscle Arnt-like protein-1) complex [6], and REV-ERBα then feeds back to inhibit the transcription of both *BMAL1* [7] and *CLOCK* [8]. Via interactions with UCP1 (uncoupling protein 1), REV-ERBα also regulates body temperature and enzymes involved in gluconeogenesis [9]. Furthermore, REV-ERBα interacts with apolipoproteins to regulate cholesterol metabolism [10]. Double-knockout of both REV-ERBs in mice leads to major disruptions in both circadian rhythms and lipid homeostasis [5].

Given the physiological processes governed by the REV-ERBs, there has been interest in pharmacologically targeting the REV-ERB proteins [11,12]. This has led to the discovery and study of both agonists and antagonists of REV-ERB. The agonist SR9009 has been demonstrated to show beneficial effects in both healthy and diseased model systems ranging from cancer to neuroinflammation to heart failure [13,14,15,16,17]. Similarly, the REV-ERB antagonist SR8278 [18] has been shown in mice to promote corneal repair [19], reduce fibrosis in dystrophic muscle [20], stimulate amyloid plaque deposition [21], prevent kidney injury [22,23], stabilize mood disorder [24], protect against ischemia–reperfusion lung injury [25], and slow tumor cell growth in mice [4]. Thus, these REV-ERB modulators have been reported to have favorable benefits in a variety of tissues and disease states.

Though skin exhibits robust circadian rhythms [26,27], the function of REV-ERBs in skin and keratinocytes that comprise the major cell type of skin epidermis has not been extensively examined. However, prior studies have included examinations of the effects of REV-ERB inhibition with SR8278 on cellular responses to UV radiation [28] and viral infection [29]. To better understand the genes that are regulated by REV-ERB and SR8278 in keratinocytes and other cell types, we performed RNA-seq analysis of SR8278-treated HaCaT keratinocytes and identified genes involved in cell proliferation and DNA synthesis as a major pathway impacted by SR8278. However, although SR8278 slowed cell proliferation in HaCaT cells, telomerase-immortalized diploid keratinocytes, and other cancer cell lines, we show here that this effect is independent of the REV-ERB nuclear receptor proteins.

## 2. Materials and Methods

### 2.1. Cell Culture

HaCaT keratinocytes [30] were obtained from Petra Boukamp (German Cancer Research Center, Heidelberg, Germany). HeLa, U2OS, and A549 cells were obtained from the University of North Carolina Lineberger Comprehensive Cancer Center Tissue Culture Facility (Chapel Hill, NC, USA). Telomerase-immortalized human neonatal diploid foreskin keratinocytes (N-TERTs) were obtained from James Rheinwald (Brigham and Women’s Hospital) [31]. HaCaT, HeLa, U2OS, and A549 cells were cultured and maintained in DMEM (Cytiva SH30243.01, Marlborough, MA, USA) containing 10% FBS (Cytiva SH30109.03, Marloborough, MA, USA), an additional 2 mM L-glutamine (Gibco 25030-081, Grand Island, NY, USA), 10,000 U/mL penicillin, and 10,000 µg/mL streptomycin (Gibco 10378-016, Grand Island, NY, USA). N-TERTs were grown in EpiLife medium (Gibco MEP1500CA, Grand Island, NY, USA) with human keratinocyte growth supplement (HKGS) (Gibco S-001-5, Grand Island, NY, USA) and penicillin/streptomycin. REV-ERBα and β-knockout (REV-ERBα/β-KO) HaCaT cells were generated by transfecting HaCaT cells with plasmids expressing Cas9 and guide RNAs targeting either REV-ERBα or REV-ERBβ and a homology template (Santa Cruz sc-401211, sc-401211-HDR, sc-402616 and sc-401616-HDR, Dallas, TX, USA), selection with puromycin, and expansion of single-cell clones. Double-KO (DKO) cells were generated by co-transfecting REV-ERBα-KO cells with the REV-ERBβ CRISPR plasmids along with pcDNA3 and then selection with geneticin (Gibco 10131-035, Grand Island, NY, USA). Transient knockdown of REV-ERBs was achieved using two transfections of siRNAs (20 nM final) targeting both REV-ERBs (sc-61458 and sc61-456) or a control siRNA (sc-37007) from Santa Cruz Biotechnology (Dallas, TX, USA) and Lipofectamine RNAiMax (Invitrogen, Waltham, MA, USA). Cells were treated with the DMSO vehicle (0.02–0.1%) (Fisher BP231-1, Fair Lawn, NY, USA) or with the indicated concentrations of SR8278 (Sigma S9576; St. Louis, MO, USA) diluted from a 50 mM stock in DMSO. Cells were exposed to UVB radiation as previously described [28].

### 2.2. Assays of Cell Survival

Methylthiazolyldiphenyl-tetrazolium bromide (MTT; Sigma M2128, St. Louis, MO, USA) assays were used to monitor cell viability/proliferation by adding the MTT reagent to cell culture medium at a final concentration of 0.25 mg/mL, incubating for 30–45 min, and then solubilizing the samples in DMSO for measurement of absorbance at 570 nm on a Synergy H1 spectrophotometer (Bio-Tek, Winooski, VT, USA). Increases in relative cell number were also determined by staining cells with crystal violet after various periods of time, solubilizing the dye in 1% SDS (Sigma L3771; St. Louis, MO, USA), and measuring the absorbance at 535 nm.

### 2.3. Flow Cytometry

HaCaT cells were treated for 2 days with 0.1% DMSO or 50 µM and then trypsinized, washed in PBS, and fixed for at least 1 day in 70% ethanol at −20 °C. Fixed cells were pelleted, washed in PBS, and then stained for 30 min in PBS containing 10 µg/mL RNase A (ThermoScientific EN0531; Rockford, IL, USA) and 0.1 mg/mL propidium iodide (Sigma P4170, St. Louis, MO, USA). Cells were analyzed on a Cytek Muse Micro flow cytometer (Freemont, CA, USA), and data were analyzed at https://floreada.io/ to gate for single cells and estimate cell cycle distribution. Experiments were performed with 4 independent biological replicate samples.

### 2.4. RNA Analyses

Cell pellets from treated cells were placed on ice, homogenized in TriZol (Ambion 15596018; Austin, TX, USA), extracted with phenol, and then purified using RNeasy columns (Qiagen 74034; Hilden, Germany). RNA was reverse transcribed using a QuantiTect Reverse Transcription Kit (Qiagen 205313; Hilden, Germany). Library preparation and Illumina sequencing were performed by Azenta Life Sciences (Burlington, MA, USA). PCRs were prepared using 2X TaqMan Fast Universal PCR Master Mix (Applied Biosystems 4444557, Carlsbad, CA, USA) and TaqMan probes (Applied Biosystems) targeting *E2F1* (Hs00153451), *RRM2* (Hs00357247), *CCNE2* (Hs00180319), and *PCNA* (Hs00427214) were used to amplify the indicated transcript. PCRs were run on an Azure Cielo 6 real-time PCR machine (Dublin, CA, USA) using an initial 3 min melting step at 95 °C followed by 40 cycles of 95 °C for 10 s and 55 °C for 30 s. The ∆∆Ct method was used to determine fold-changes in gene expression using beta-2-microglobulin (B2M; Hs00187842) as a housekeeping gene.

### 2.5. RNA-Seq and Bioinformatic Analyses

RNA was purified from two biological replicates of HaCaT cells treated with DMSO or 50 µM SR8278 for 24 h and submitted to Azenta Life Sciences for library construction and Illumina sequencing. Between 38 and 42 million reads were present in each replicate, and each dataset had a mean quality score greater than 39. The DESeq2 software version 2.12 package was used to identify significantly differentially expressed genes (log2 fold change >1 or <−1, and *p* value < 0.05). The heatmap() function in R version 4.3.2 was used to assign a red or blue color to a gene based on whether the expression was higher or lower than the control, respectively. Additionally, the heatmap() function was used to generate a neighbor-joining tree, which grouped together like-genes based on the sort condition (log2 fold change). Gene Ontology Enrichment Analysis was used to find biological processes significantly altered because of SR8278 treatment. The RNA-seq data is available at NCBI’s Sequence Read Archive (SRA) under BioProject accession number PRJNA1302391.

### 2.6. Protein Immunoblotting

Cells were lysed in either 1X SDS-PAGE sample buffer or ice-cold RIPA buffer (ThermoScientific J62725.AP), and then soluble protein lysates were separated on Tris-Glycine SDS polyacrylamide gels. Proteins were then transferred to a nitrocellulose membrane (Bio-Rad 16200094, Hercules, CA, USA) using a semi-dry transfer apparatus. Blots were stained with 0.5% Ponceau S (Sigma P3504, St. Louis, MO, USA) to ensure equal loading. The blots were blocked in 5% non-fat milk (Meijer, Tipp City, OH, USA) in TBST (Tris-buffered saline containing 0.1% Tween-20) and then probed overnight with primary antibodies from Cell Signaling Technology (Danvers, MA, USA) recognizing E2F1 (#3742), RRM2 (#65939), Cyclin E2 (#4132), phospho-CHK1 (Ser345; #2348), phospho-H2AX (Ser1139; #9718), PARP (#9542), or REV-ERBα (#13418), antibodies from Santa Cruz Biotechnology (Dallas, TX, USA) recognizing PCNA (sc-56) or REV-ERBβ (sc-398252), or an antibody from Bethyl recognizing phospho-KAP1 (Ser824; A300-767A). After washing with TBST, blots were probed with HRP-coupled anti-rabbit or anti-mouse IgG (Invitrogen) secondary antibodies for one hour at room temperature. Chemiluminescence was visualized with Clarity Western ECL substrate (Bio-Rad 1705061, Hercules, CA, USA) using an Azure 600 Western blot imager (Dublin, CA, USA). Signals in the linear range of detection were quantified by densitometry using Image Lab software version 6.1 (Bio-Rad, Hercules, CA, USA) and normalized to the Ponceau S-stained membranes.

### 2.7. Statistical Analyses

GraphPad Prism version 10 was used for all data analyses. Unless otherwise indicated, data were normally distributed as measured by Shapiro–Wilk test. Two-way or one-way ANOVAs, paired Student’s *t*-tests, and one-sample *t*-tests were used to compare treatment groups, and the test that was used along with appropriate post hoc test is described in the figure legend.

## 3. Results

### 3.1. SR8278 Impacts the Expression of Genes Involved in Cell Proliferation

**Figure 1:**
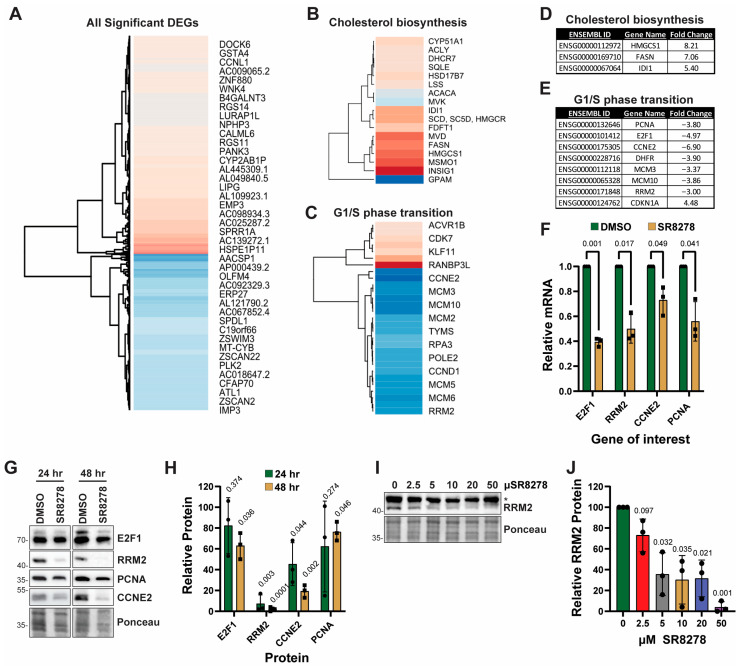
Impact of SR8278 on gene expression in HaCaT keratinocytes: (**A**) HaCaT cells are treated with 0.1% DMSO (dimethylsulfoxide) or 50 µM SR8278 for 24 h, and then RNA is purified and subjected to RNA-seq analysis. Differentially expressed genes (DEGs) are graphed and sorted based on the level of log2 fold-change in gene expression, and a red or blue color is assigned to genes based on whether the expression was higher or lower than the DMSO control, respectively. And a neighbor-joining tree was assembled to group genes based on expression. Gene Ontology Enrichment Analysis is used to find biological processes significantly altered because of SR8278 treatment. Two of the most significantly (*p* < 0.05) changed pathways are included. (**B**) Genes that contribute to the regulation of cholesterol biosynthetisis and (**C**) G1/S transition of mitotic cell cycle are highlighted. (**D**,**E**) Specific genes of interest within the two biological processes that were the most significantly altered (*p* < 0.00001) are shown. (**F**) RT-qPCR is used to analyze the relative expression of the indicated genes at the mRNA level in cells treated with DMSO or SR8278 for 24 h. Results show the relative expression from each of three independent biological replicate experiments along with the average and standard deviation. One-sample *t*-tests comparing to a hypothetical value of 1.0 (the ΔΔCt values for DMSO-treated cells) are used to make comparisons between the SR8278- and DMSO-treatment groups (*p*-values are shown above the bars in the graph. (**G**) Lysates from cells treated for 24 or 48 h with DMSO or SR82798 are subjected to Western blot analysis. (**H**) Quantitation of three independent biological replicate experiments performed as in (**G**) and analyzed by one-sample *t*-tests. (**I**) Lysates from cells treated with increasing concentrations of SR8278 for 48 h are analyzed by Western blotting. The asterisk (*) indicates a non-specific cross-reacting band. (**J**) Quantitation of three independent biological replicate experiments performed as in (**I**) and analyzed by a one-sample *t*-test.

Though the small molecule SR8278 is reported to be an inhibitor of the REV-ERB nuclear receptor proteins [18], the genes impacted by SR8278 treatment in human keratinocytes have not been examined. Because of our prior work showing that SR8278 at a concentration of 50 µM maximally protected HaCaT keratinocytes in vitro from the negative effects of UVB radiation [28], we treated HaCaT keratinocytes with either vehicle (0.1% DMSO) or 50 µM SR8278 for 24 h and then total RNA was subjected to RNA-seq analysis to identify genes differentially affected by SR8278 treatment. A total of 2686 genes met the threshold criteria to be classified as significantly altered by SR8278 treatment (log2 fold change >1 or <−1, and *p* value < 0.05), and a subset of the most differentially affected genes is shown in Figure 1A. Gene ontology enrichment analysis indicated that the genes could be classified into two major biological processes, including the regulation of cholesterol biosynthesis (Figure 1B) and of the G1/S phase transition of the mitotic cell cycle (Figure 1C). The most significantly altered genes in each of these two biological pathways are shown in Figure 1D,E.

Because of our interest in cell growth and proliferation, we used TaqMan-based RT-qPCR to confirm a subset of the genes identified with the RNA-seq approach. As shown in Figure 1F, SR8278 induced a significant decrease in the expression of several genes involved in the G1/S phase transition and cell proliferation, including the pro-S phase transcription factor *E2F1*, the dNTP-synthesizing gene *RRM2*, the cyclin-dependent kinase regulator *Cyclin E2* (*CCNE2*), and the DNA synthesis factor *PCNA*. Western blot analysis further confirmed that SR8278 treatment for 24 or 48 h led to reduced expression of these genes at the protein level (Figure 1G; original blots are shown in Supplementary Figures S1 and S2).

Cell culture studies involving SR8278 have traditionally used the drug at a concentration of 5–10 µM. To determine whether lower concentrations of SR8278 impact the expression of proliferation proteins, HaCaT cells were treated with a range of concentrations of SR8278 for 48 h. As shown in Figure 1I,J (original blots are shown in Supplementary Figure S3), SR8278 induced a clear dose-dependent decrease in RRM2 protein levels. SR8278 at a concentration as low as 5 µM was sufficient to induce a statistically significant reduction in RRM2 protein levels.

As our RNA-seq data indicated that the expression of the cyclin-dependent kinase inhibitor *CDKN1A/p21* was increased in SR8278-treated cells, we also examined whether p21 protein levels were elevated. However, as shown in Supplementary Figure S4, p21 protein levels were not found to be increased by SR8278 treatment.

### 3.2. SR8278 Slows Cell Proliferation in Multiple Cell Lines

**Figure 2:**
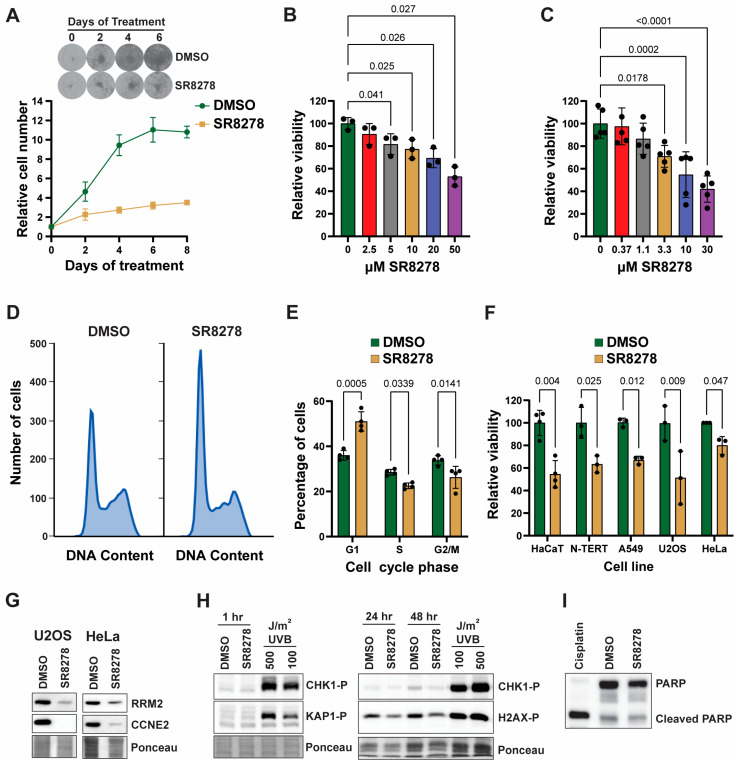
SR8278 slows cell proliferation but does not induce genotoxic stress or cell death: (**A**) A representative experiment in which HaCaT cells are treated with DMSO (0.1%) or 50 µM SR8278 and then stained with crystal violet on the indicated days to visualize cell number and cell growth. Quantitation of results (average and standard deviation) from 3 independent experiments. (**B**) MTT assays are used to monitor relative cell viability 4 days after treating HaCaT cells with the indicated concentration of SR8278. Results show the average and standard deviation from 3 independent experiments. A one-way ANOVA with a Dunnett’s multiple comparisons post hoc test is used to compare the treatment groups to the untreated cells. (**C**) MTT experiments are performed as in (**B**) but with telomerase-immortalized diploid N-TERT keratinocytes treated for 3 days. (**D**) HaCaT cells are treated as in (**A**) for 2 days, and then fixed cells are stained with propidium iodidine to measure DNA content by flow cytometry. (**E**) Cell cycle distribution is estimated by DNA content from 4 independent experiments performed as in (**D**). A two-way ANOVA with Tukey’s multiple comparisons test is used to compare the percentage of cells in each of the cell cycle phases in the two treatment groups. (**F**) The indicated cell lines are treated with DMSO or SR8278 (10 µM for N-TERT cells but 50 µM for the other cell lines) for between 3 and 9 days, and then MTT assays from 3 to 4 independent experiments are performed to monitor relative cell viability. Paired Student’s *t*-tests using the Holm–Šidák method are used to compare the treatment groups in the HaCaT, N-TERT-, and U2OS cells. For the A549 and HeLa cells, one of each of the treatment groups does not show a normal distribution as determined by the Shapiro–Wilk test, and, thus, a one-sample *t*-test is used to compare cell viability to a control value of 100. (**G**) Cell lysates from U2OS or HeLa cells treated with DMSO or SR8278 for 2 days are subjected to Western blotting. (**H**) HaCaT cells are treated as in A but are harvested at the indicated time points for analysis of DNA damage checkpoint signaling by Western blotting. Cells are harvested 1 h after exposure to the indicated fluence of UVB radiation as a positive control. (**I**) Western blotting is used to detect PARP (poly [ADP-ribose] polymerase) cleavage as a measure of apoptosis in cells treated for 24 h with 30 µM cisplatin, 0.1% DMSO or 50 µM SR8278. All bar graphs indicate the average and standard deviation.

To determine whether the reduced expression of these gene products by SR8278 treatment is correlated with slower cell growth, we treated HaCaT keratinocytes with 50 µM SR8278 and then visualized cell growth by staining cells with crystal violet. As shown in Figure 2A, SR8278 significantly inhibited cell growth and proliferation. MTT assay further revealed a dose-dependent decrease in cell proliferation after 4 days of treatment in both HaCaT cells (Figure 2B) and telomerase-immortalized, diploid N-TERT keratinocytes (Figure 2C). Consistent with the reduced expression of proteins needed for proliferation and cell cycle progression, analysis of cell cycle distribution based on DNA content of propidium iodide-stained HaCaT cells indicated an increased number of cells in G1 phase and a reduced number of cells in S and G2/M phase in cells treated with SR8278 relative to cells treated with DMSO vehicle (Figure 2D,E).

To determine whether SR8278 slows the rate of cell proliferation in cells other than keratinocytes, we repeated MTT assays using not only HaCaT and N-TERT keratinocytes but also A549 lung carcinoma, U2OS osteosarcoma, and HeLa cervical cancer cells treated with either vehicle or DMSO. SR8278 treatment resulted in lower cell proliferation in all tested cell lines (Figure 2F). Similarly, Western blot analysis showed that SR8278 induced significant reductions in the expression of both RRM2 and Cyclin E in both USOS and HeLa cells (Figure 2G; original Western blots are shown in Supplementary Figure S5). Decreased expression of RRM2 and other proliferation proteins could lead to the activation of DNA damage and replication checkpoint signaling cascades. However, we did not observe any phosphorylation of CHK1, KAP1, or H2AX, which suggests that SR8278 is not genotoxic (Figure 2H; original blots are shown in Supplementary Figure S6). Moreover, we did not observe any obvious effects of SR8278 on cell death, such as floating cells in the culture media. We nonetheless investigated whether the reduced cell proliferation is associated with increased apoptosis but did not detect any evidence of caspase activation by SR8278 (Figure 2I; original Western blots are shown in Supplementary Figure S7). Thus, the anti-proliferative effect of SR8278 does not appear to be due to genotoxic stress or the induction of apoptosis.

### 3.3. SR8278 Slows Cell Proliferation Independent of REV-ERB

**Figure 3:**
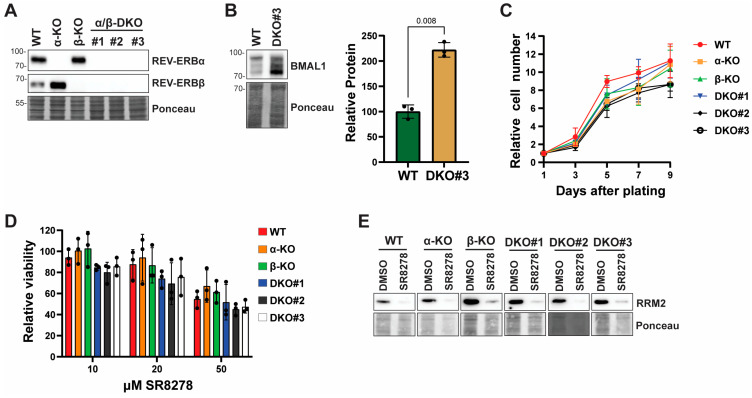
SR8278 slows cell proliferation independent of the REV-ERB proteins in HaCaT keratinocytes: (**A**) The expression of the REV-ERB (reverse c-erbAa) proteins is examined in cell lysates from wild-type (WT) HaCaT cells and REV-ERBα, REV-ERBβ, and REV-ERBα/β double-knockout (DKO) HaCaT cell lines generated with CRISPR/Cas9 genome editing. (**B**) Lysates from WT and REV-ERB DKO#3 cells are examined for BMAL1 protein expression by Western blotting. The graph shows the average and standard deviation from three independent sets of lysates, which are analyzed by a paired *t*-test. (**C**) The WT and REV-ERB KO cell lines are plated and then stained with crystal violet at the indicated times to quantify cell growth and proliferation. Results show the average and standard deviation from three independent experiments. (**D**) The six cell lines are treated with increasing concentrations of SR8278 for 3 days, and then MTT assays are performed to monitor cell proliferation. Results show the individual values from three independent experiments along with the average and standard deviation. A two-way ANOVA with Tukey’s post hoc test is used to compare the viability of each of the KO cell lines with WT HaCaT cells but fails to find any significant differences (*p* > 0.05 for all comparisons), regardless of SR8278 treatment concentration. (**E**) Cells are treated with DMSO or SR8278 for 48 h, and then Western blotting is carried out for RRM2. The relative protein expression is indicated below the immunoblots.

To provide genetic evidence that SR8278 acts via either of the two REV-ERB proteins (REV-ERBα or REV-ERBβ), we used CRISPR/Cas9 genome editing to generate HaCaT cell lines lacking expression of one or both REV-ERB proteins. As shown in Figure 3A, Western blot analysis showed that a single knockout of each REV-ERB protein was associated with a corresponding increase in the expression of the other REV-ERB protein (original Western blots are shown in Supplementary Figure S8). Furthermore, we were also able to generate REV-ERBα/β double-knockout (DKO) HaCaT cell lines (Figure 3A). To validate that loss of the REV-ERB proteins has functional effects related to the loss of REV-ERB activity, we examined the protein levels of the REV-ERB target gene BMAL1. As shown in Figure 3B, BMAL1 protein levels were found to be approximately 2.2-fold higher in one of the knockout cells in comparison to wild-type HaCaT cells (original blots are shown in Supplementary Figure S9).

Though the pharmacological REV-ERB antagonist SR8278 slowed cell growth in both HaCaT and other cell lines (Figure 2), we noted no significant differences in cell growth rates between the single- and double-REV-ERB knockout cell lines (Figure 3C), indicating that genetic loss of REV-ERB does not impact the growth rate of HaCaT keratinocytes. Moreover, when we treated the single- and double-knockout cells with different concentrations of SR8278, we observed a similar inhibition of cell proliferation after 3 days of treatment (Figure 3D). Consistent with these results, SR8278 caused a similar decrease in E2F1 and RRM2 protein expression in all the cell lines (Figure 3E; original Western blots are shown in Supplementary Figure S10). We conclude that the effect of SR8278 on cell proliferation is independent of the REV-ERB proteins in HaCaT keratinocytes.

**Figure 4:**
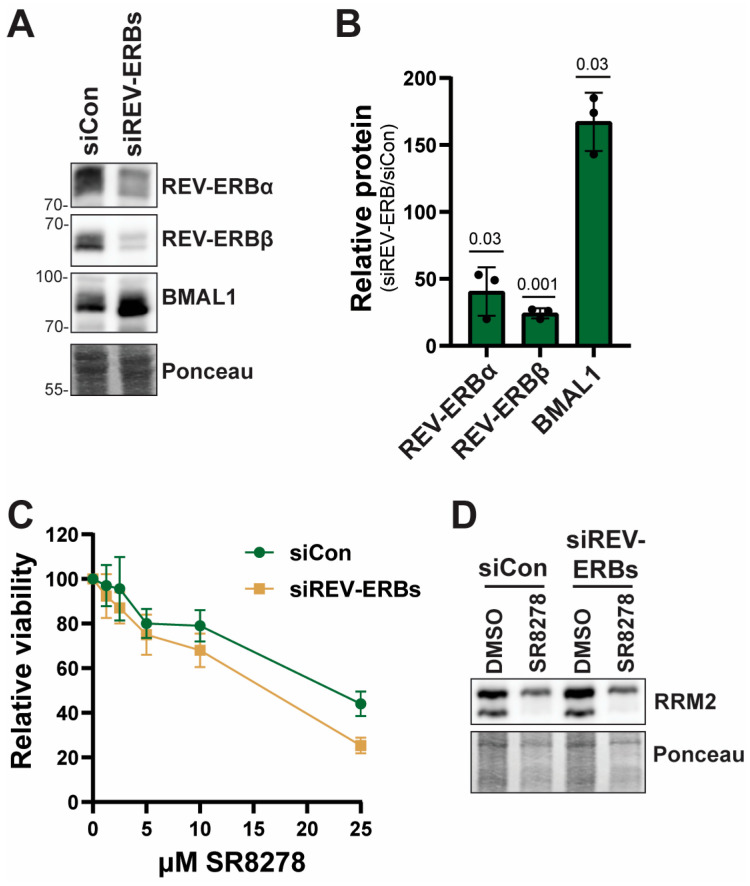
SR8278 slows cell proliferation independent of the REV-ERB proteins in N-TERT keratinocytes: (**A**) N-TERT keratinocytes are transfected twice with 40 nM control siRNA (siCon) or 20 nM of each of siRNAs targeting REV-ERBα and REV-ERBβ. Lysates are prepared 24 h after the last transfection and subjected to Western blotting for the indicated proteins. (**B**) Quantitation of the indicated proteins in cells transfected with REV-ERB siRNAs versus control siRNA from three biological replicate experiments. Differences are examined by one-sample *t*-tests compared to a hypothetical value of 100 (no change in expression). (**C**) Following transfection, cells in (**A**) are treated with the indicated concentration of SR8278 for 4 days, and then MTT assays are performed. The results are the average and standard deviation from three independent experiments. (**D**) Lysates from cells treated as in (**C**) are harvested 48 h after treatment with 0.05% DMSO or 25 µM SR8278 and examined by Western blotting.

To provide additional support that the effect of SR8278 on cell proliferation is independent of the REV-ERB proteins, we knocked down REV-ERBα and REV-ERBβ in N-TERT keratinocytes by transfecting cells with either a control siRNA or siRNAs targeting the *REV-ERB mRNAs*. As shown in Figure 4A,B, expression of both REV-ERB proteins was reduced to a significant extent by the *REV-ERB* siRNAs. Moreover, expression of the REV-ERB target BMAL1 was elevated by approximately 1.6-fold by REV-ERB disruption (Figure 4A,B; original blots are shown in Supplementary Figure S11), indicating that the loss of REV-ERB expression has functional effects in the cells.

We next treated the transfected cells with increasing concentrations of SR8278 and then monitored cell viability 4 days later using MTT assays. As shown in Figure 4C, SR8278 induced a similar dose-dependent decrease in cell viability in cells transfected with either control or REV-ERB siRNAs. Furthermore, Western blotting showed that SR8278 treatment caused a decrease in RRM2 protein expression in cells regardless of the status of REV-ERB expression (Figure 4D; original blots are shown in Supplementary Figure S12).

## 4. Discussion

Related to our findings here on the effects of SR8278 on slowing keratinocyte proliferation in vitro, recent work has also shown that SR8278 slows tumor growth in mice in vivo and is correlated with effects on the expression of genes involved in diverse growth factor signaling pathways [4]. Thus, SR8278 appears to be able to slow cell proliferation in a variety of different cell types both in vitro and in vivo. Though we observed that SR8278 clearly slowed cell proliferation in both keratinocyte and a few cancer cell lines in vitro (Figure 2) and negatively affected the expression of several different proliferation genes (Figure 1), we found that genetic disruption of the REV-ERBs had no impact on cell growth and proliferation in both HaCaT and N-TERT keratinocytes (Figure 3 and Figure 4), which suggests that the growth inhibitory effect of SR8278 was not mediated by its effects on the REV-ERB proteins.

In contrast, using lentiviral gRNA and shRNA/siRNA approaches, Yang et al. recently showed that knockdown of REV-ERBα expression alone slowed the growth of several cancer cell lines that display disrupted circadian rhythmicity in vitro but had little-to-no effect on cell lines with an intact circadian rhythm [4]. Moreover, Yang et al. reported that transient REV-ERBα knockdown led to the induction of apoptosis. However, we were able to readily generate stable REV-ERB single- and double-knockout HaCaT cells (Figure 3A), which have an intact circadian clock [32], and did not find that SR8278 induces apoptosis (Figure 2I). Moreover, we found that SR8278 slows proliferation in circadian clock-intact N-TERT keratinocytes [29] even when REV-ERB expression is transiently but significantly reduced (Figure 4). The reason for these differences between our studies and that of Yang et al. remains to be determined but could be due to acute knockdown of only REV-ERBα or β in clock-disrupted cancer cells, which may exhibit different effects than dual knockdown or knockout in the clock-intact keratinocytes used in our work here. Thus, it may be important to compare transient knockdown versus stable knockout in other cell lines to better understand the role of REV-ERBs in cell proliferation. Nonetheless, we note that REV-ERBα/β double-knockout mice have been generated [33], which suggests that REV-ERB is not essential for cell proliferation during mouse development. However, the requirement for REV-ERB may be different in tumor cells.

As described in the introduction above, SR8278 has been reported to exert beneficial effects in a variety of experimental systems and pathological conditions and is assumed to be mediated by its effects on REV-ERBα and/or β. However, most previous studies involving SR8278 have not used genetic REV-ERB knockdown or knockout approaches to show that REV-ERB loss acts in a similar manner as the purported REV-ERB inhibitor SR8278. Our data here suggests that this may be an important control to validate that the effects of REV-ERB modulators indeed act via REV-ERB.

The mechanism of the REV-ERB-independent effects of SR8278 on cell proliferation remains to be determined. Though many of the effects observed in our work here used concentrations (50 µM) that are approximately 5-fold higher than commonly used in many other cell-based studies of SR8278 (5–10 µM), our data show that even lower concentrations partially alter the expression of DNA replication proteins (Figure 1I,J) and slow keratinocyte growth (Figure 2B,C). Interestingly, the REV-ERB agonist SR9009 has similarly been reported to inhibit cell proliferation independent of REV-ERB using cell lines derived from mice [33]. Thus, our results showing that SR8278 inhibits cell proliferation independent of REV-ERB may suggest a limitation with the family of compounds shown to target REV-ERB at least as they relate to effects on cell proliferation. The exact target(s) of SR9009 and SR8278 that are relevant to their effects on cell proliferation remain to be determined and may be a worthwhile investigation given the increasing number of studies using these compounds. Though our data and that of Dierickx et al. raise concerns about the interpretation of results when using SR8278 and SR9009 in studies of cell proliferation, it remains possible that the effects of SR8278 on cell proliferation only maximally occur at high concentrations that target some other unknown factor. Thus, SR8278 and SR9009 may still be useful tools to pharmacologically modulate REV-ERB function, but care should be taken to use complementary genetic approaches to validate key findings.

## 5. Conclusions

Based on our data presented here, we suggest that care should be taken when interpreting the results of cell proliferating experiments using SR8278 that lack complementary genetic knockdown or knockout approaches due to potential off-target effects of the compound.

## Data Availability

All data generated or analyzed during this study are available at Mendeley Data (doi: 10.17632/vzr4snpdb4.1). The RNA-seq data has been deposited at NCBI’s Sequence Read Archive (SRA) under BioProject accession number PRJNA1302391.

## Supplementary Materials

The following supporting information can be downloaded at: https://www.mdpi.com/article/10.3390/biom16030416/s1, Figure S1: Original Western blots and experimental replicates for Figure 1G (24 h); Figure S2: Original Western blots and experimental replicates for Figure 1G (48 h); Figure S3: Original Western blots and experimental replicates for Figure 1I; Figure S4: SR8278 treatment does not lead to increased p21 protein levels in HaCaT cells; Figure S5: Original Western blots and experimental replicates for Figure 2G; Figure S6: Original Western blots and experimental replicates for Figure 2H; Figure S7: Original Western blots and experimental replicates for Figure 2I; Figure S8: Original Western blots and experimental replicates for Figure 3A; Figure S9: Original Western blot for Figure 3B; Figure S10: Original Western blots and experimental replicates for Figure 3E; Figure S11: Original Western blots and experimental replicates for Figure 4A; Figure S12: Original Western blots and experimental replicates for Figure 4D.

## Abbreviations

The following abbreviations are used in this manuscript:

BMAL1	Brain and muscle Arnt-like protein-1
CLOCK	Circadian locomotor output kaput
NR1D1/NR1D2	Nuclear receptor subfamily 1 group D members 1 and 2
PER1	Period1
REV-ERB	Reverse c-erbAa
ROR	Retinoic acid-like orphan receptor
