# Supplementary material for: The Small Molecule SR8278 Inhibits Cell Proliferation Independent of the REV-ERB Nuclear Receptor Proteins in Human Keratinocytes
Source: Biomolecules. 2026 Mar 12;16(3):416. doi: 10.3390/biom16030416 (PMC13024357; doi:10.3390/biom16030416)
Supplement: Supplementary file 1 [file biomolecules-16-00416-s001.zip › biomolecules-4113897-supplementary.pdf]

## SUPPLEMENTARY DATA

All data generated or analyzed during this study are available at Mendeley Data (doi: 10.17632/vzr4snpdb4.1).

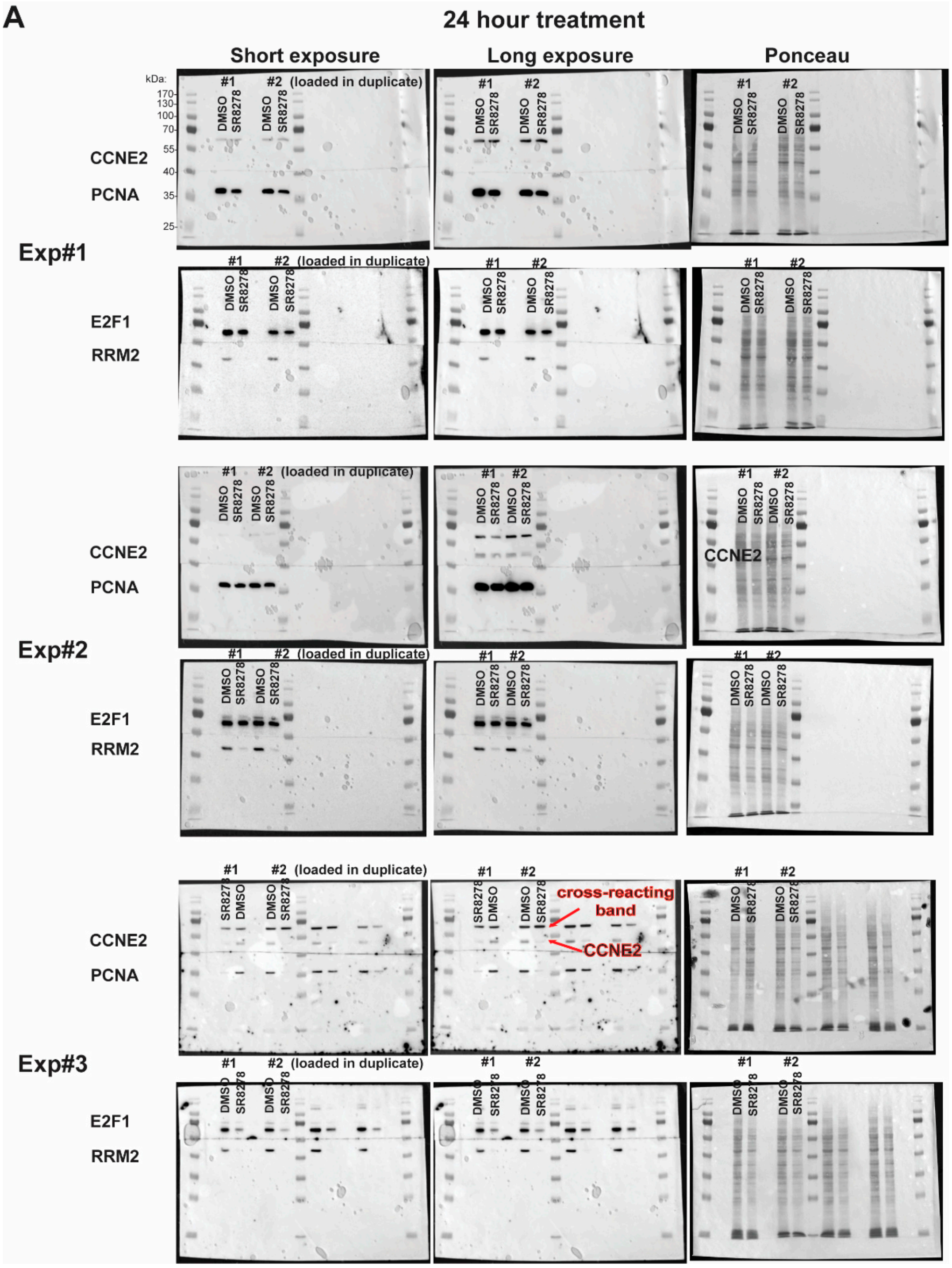


**Supplementary Figure S1. Original western blots and experimental replicates for Figure 1G (24 hr).** HaCaT cells were treated with DMSO or 50 μM SR8278 for 24 hours. The data show the original, uncropped western blots from three independent experiments (Exp#1-3), which is summarized in Figure 1G. Chemiluminescent signals were merged with a white light image of the blots to show the location of the indicated proteins relative to the molecular weight markers.

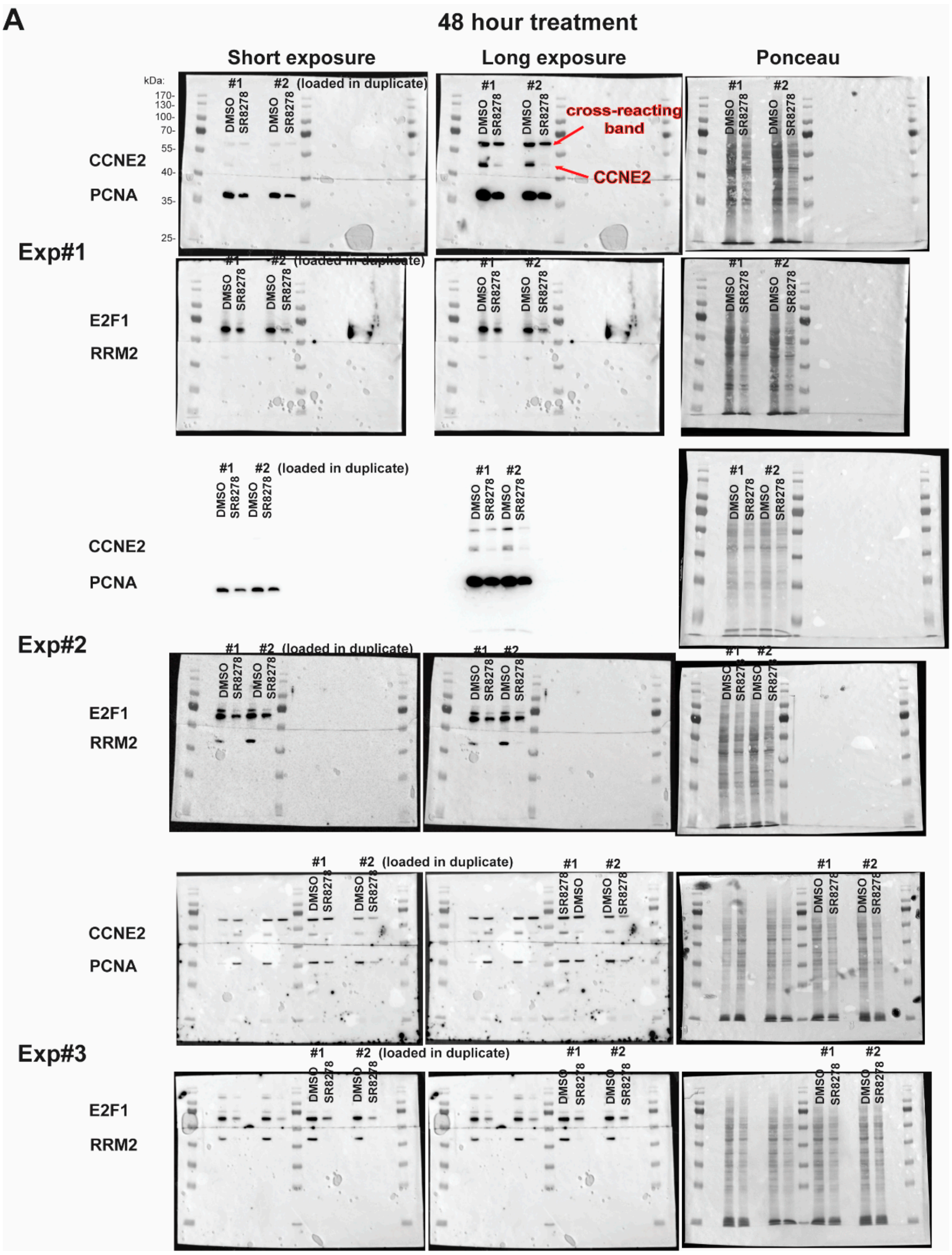


**Supplementary Figure S2. Original western blots and experimental replicates for Figure 1G (48 hr).** HaCaT cells were treated with DMSO or 50 µM SR8278 for 48 hours. The data show the original, uncropped western blots from three independent experiments (Exp#1-3), which is summarized in Figure 1G. With the exception of the CCNE2 and PCNA immunoblots for Experiment #2, chemiluminescent signals were merged with a white light image of the blots to show the location of the indicated proteins relative to the molecular weight markers.

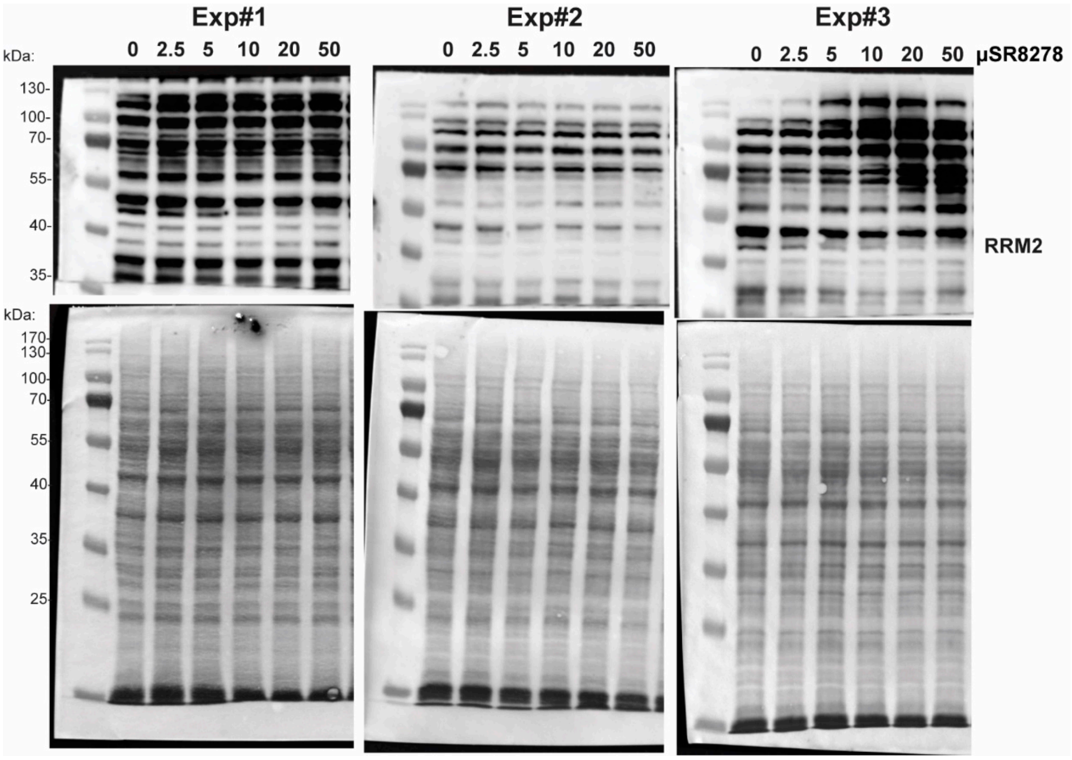


**Supplementary Figure S3. Original western blots and experimental replicates for Figure 1I.** HaCaT cells were treated with the indicated concentration of SR8278 for 48 hours. The data show the original, uncropped western blots from three independent experiments (Exp#1-3), which is summarized in Figure 1I-J. Chemiluminescent signals were merged with a white light image of the blots to show the location of the indicated proteins relative to the molecular weight markers. Many more cross-reacting bands were noted on these blots in comparison to the blots in Supplementary Figure 1-2, which may be due to a combination of the RRM2 antibody getting old and the use of a new anti-rabbit secondary antibody.

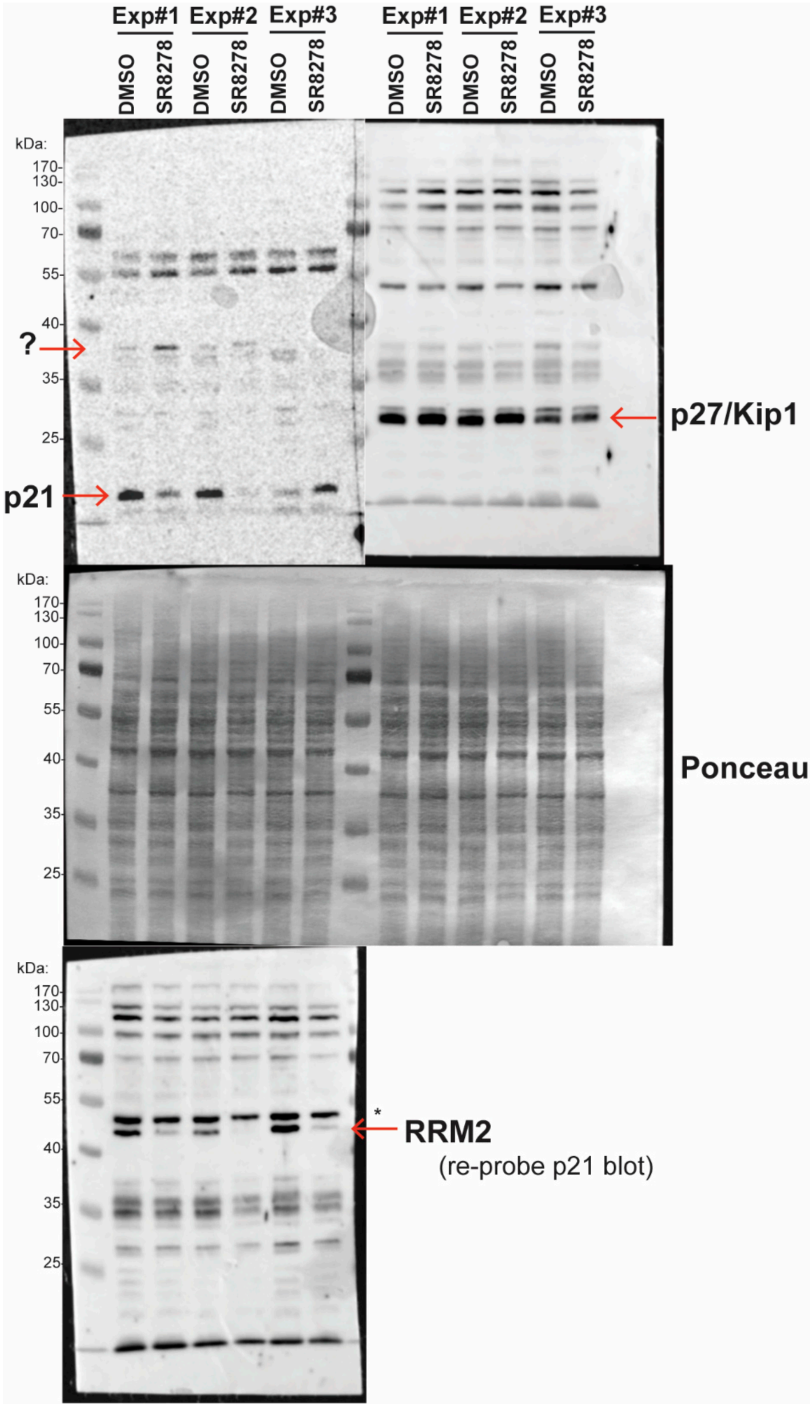


**Supplementary Figure S4. SR8278 treatment does not lead to increased p21 protein levels in HaCaT cells.** HaCaT cells were treated with 0.1% DMSO or 50 μM SR8278 for 48 hours, and then cell lysates were examined by western blotting. The data show the original, uncropped western blots from three independent experiments (Exp#1-3). On the p21 blot, a band of approximately 37 kDa was observed that appeared to be increased in SR8278-treated cells relative to DMSO-treated cells.

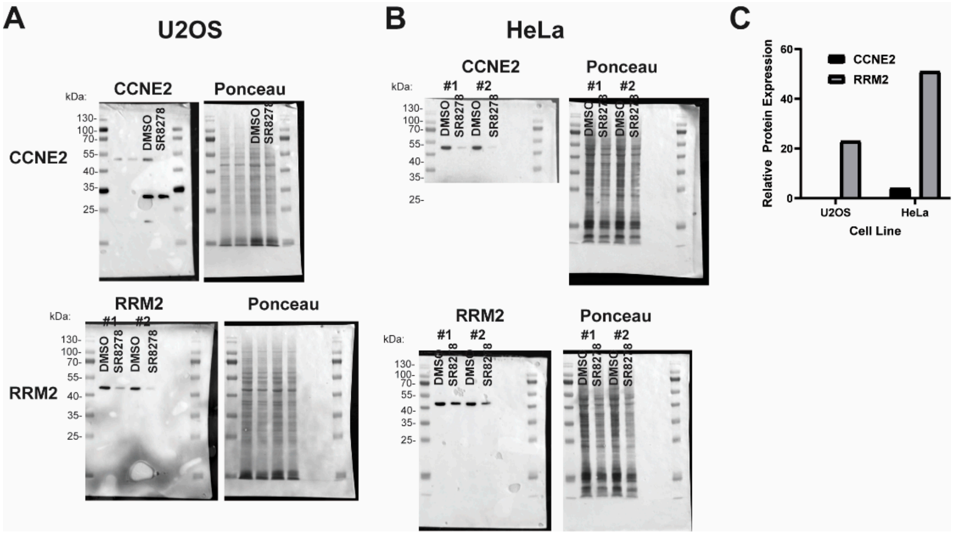


**Supplementary Figure S5. Original western blots and experimental replicates for Figure 2G. (A)** U2OS cells were treated with DMSO or 50 μM SR8278 for 48 hours. Whole cell lysates were prepared for western blot analysis of CCNE2 and RRM2. These images show the full, uncropped western blots shown in Figure 2E. **(B)** HeLa cells were treated as in (A). **(C)** Quantitation of western blots in (A) and (B) in which the indicated protein was normalized to the DMSO-treated control cells, which was set to an arbitrary value of 100.

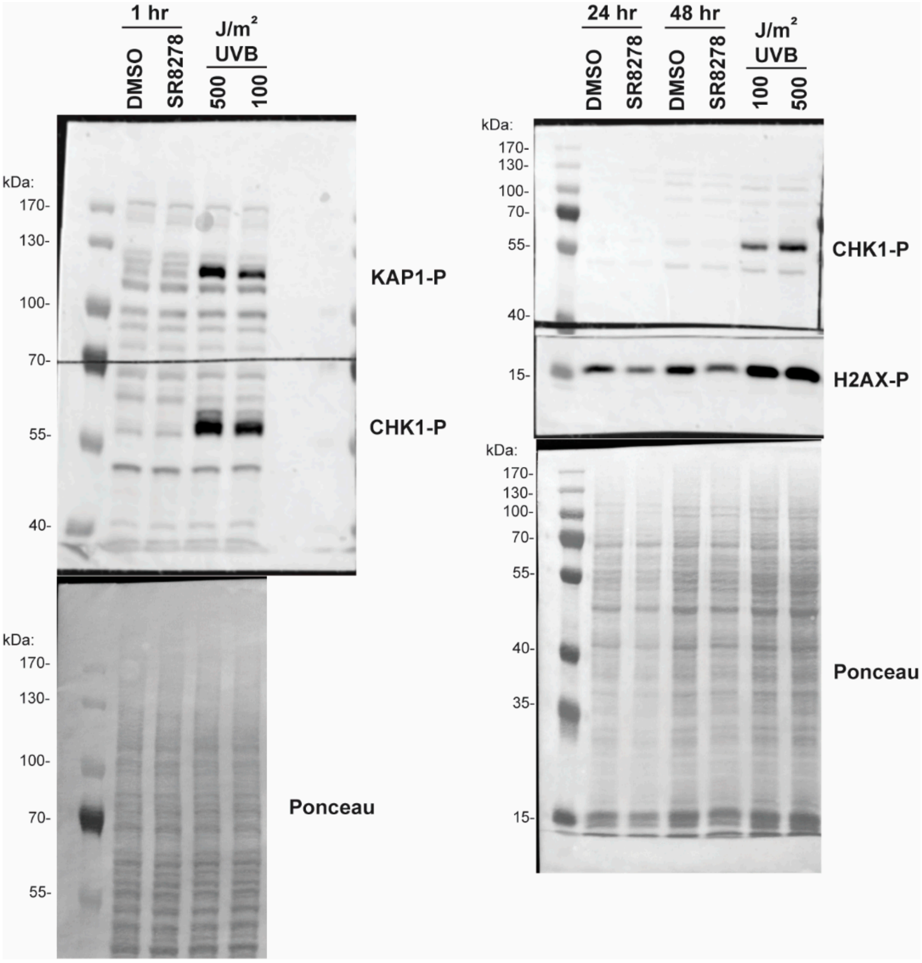


**Supplementary Figure S6. Original western blots and experimental replicates for Figure 2H.** HaCaT cells were treated with 0.1% DMSO or 50 μM SR8278 for the indicated periods of time or exposed to the indicated fluence of UVB radiation and then harvested 1 hr later. Lysates were examined by western blotting for phospho-KAP1 (Ser824), phospho-CHK1 (Ser345), or phospho-H2AX (Ser139).

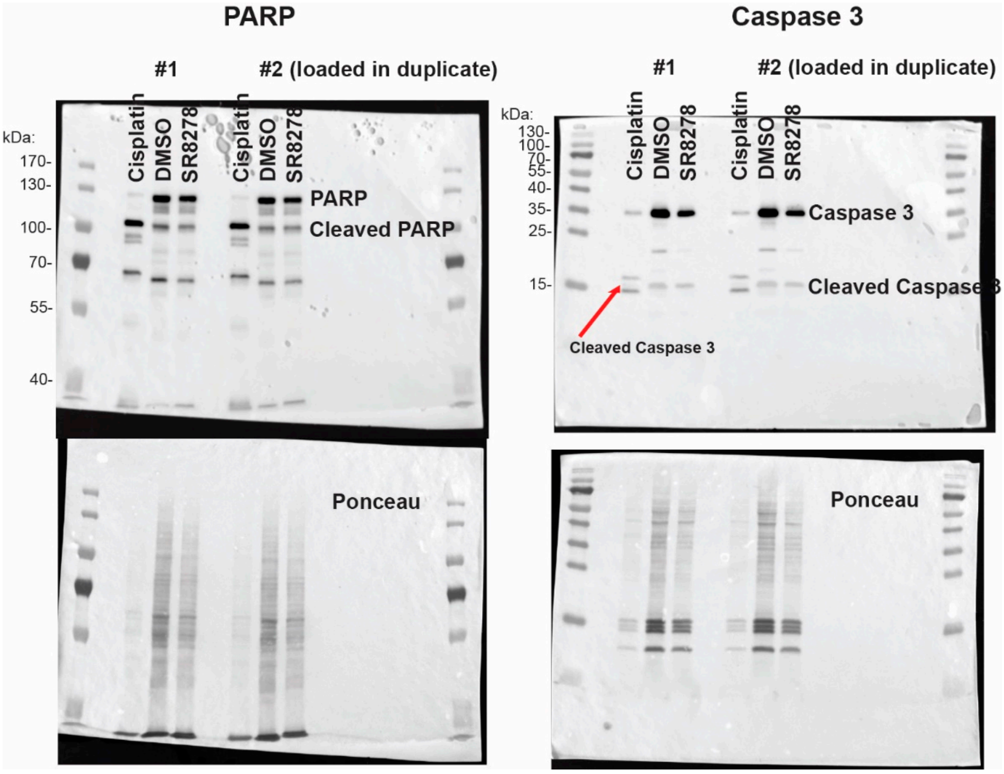


**Supplementary Figure S7. Original western blots and experimental replicates for Figure 2I. (A)** HaCaT cells were treated with 30 μM cisplatin, 0.1% DMSO, or 50 μM SR8278 for 24 hours. Whole cell lysates were analyzed by western blotting for PARP and Caspase 3. The locations of the cleaved forms of both proteins are indicated. The PARP immunoblot shows the full, uncropped image shown in Figure 2F.

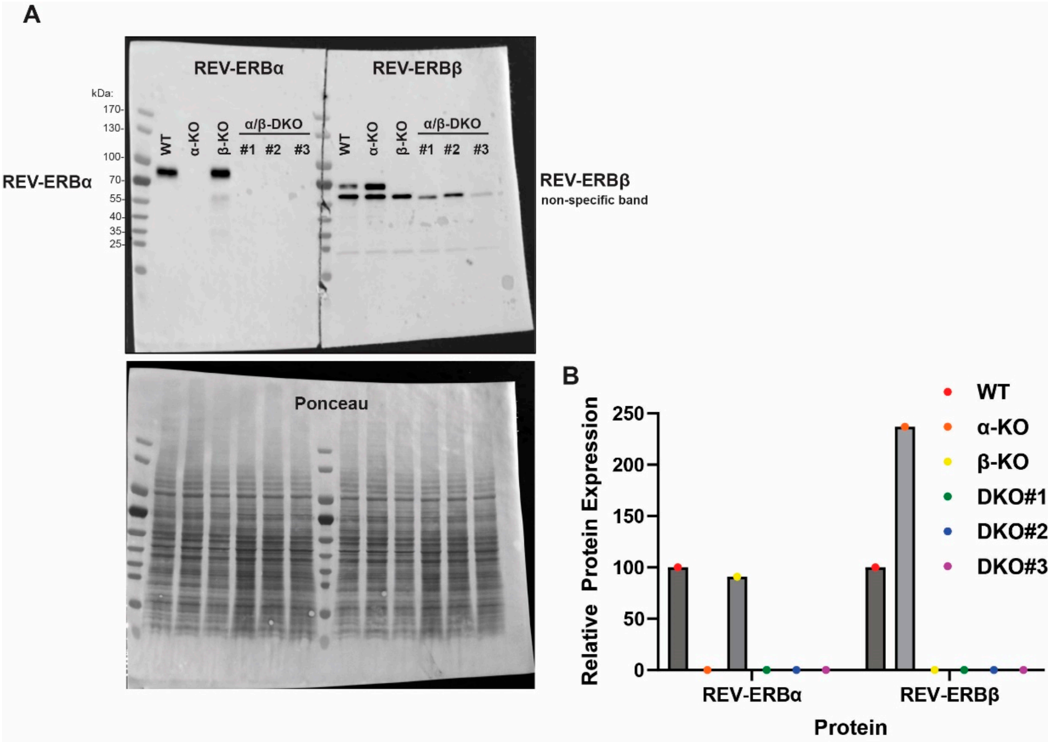


**Supplementary Figure S8. Original western blots and experimental replicates for Figure 3A. (A)** Western blot analysis of REV-ERB protein expression in the indicated cell lines. These data show the full, uncropped western blots for Figure 3A. **(B)** Densitometric quantification of western blot results from (A) in which protein expression was normalized to wild-type (WT) HaCaT cells.

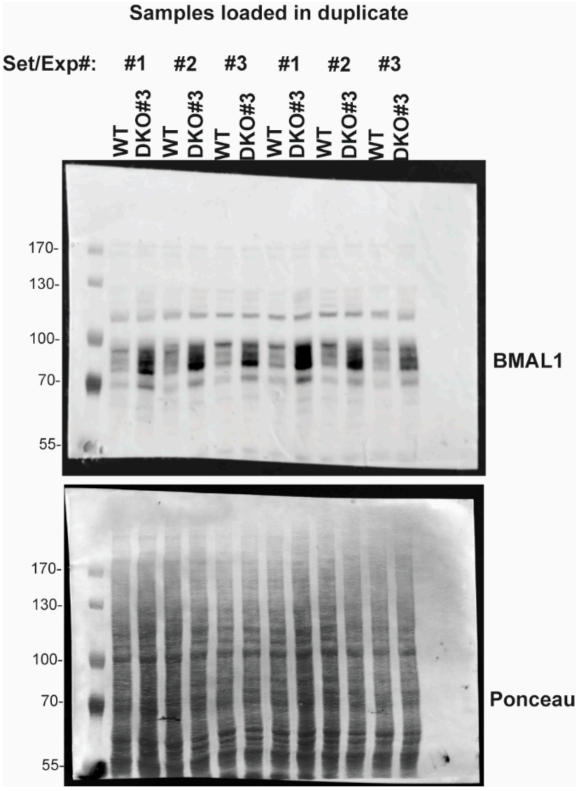


**Supplementary Figure S9. Original western blot for Figure 3B.** Lysates from three independent sets of wild-type (WT) and REV-ERB double-knockout (DKO#3) cells were loaded in duplicate on an SDS-polyacrylamide gel and then examined by Western blotting for BMAL1.

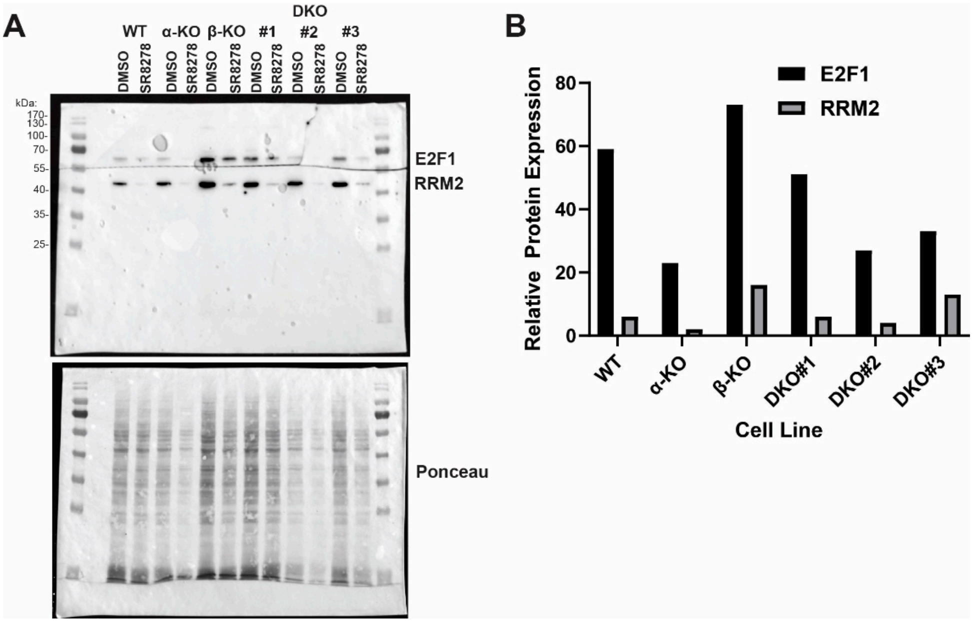


**Supplementary Figure S10. Original western blots and experimental replicates for Figure 3E.** The indicated wild-type (WT) or REV-ERB knockout (KO) HaCaT cell line was treated with DMSO or 50 μM SR8278 for 48 hours. Whole cell lysates were prepared and analyzed by western blotting. These data show the full, uncropped western blots for Figure 3D. **(B)** Densitometric analysis of the results in (A), where the protein expression was normalized to the DMSO-treated control, which was set to an arbitrary value of 100.

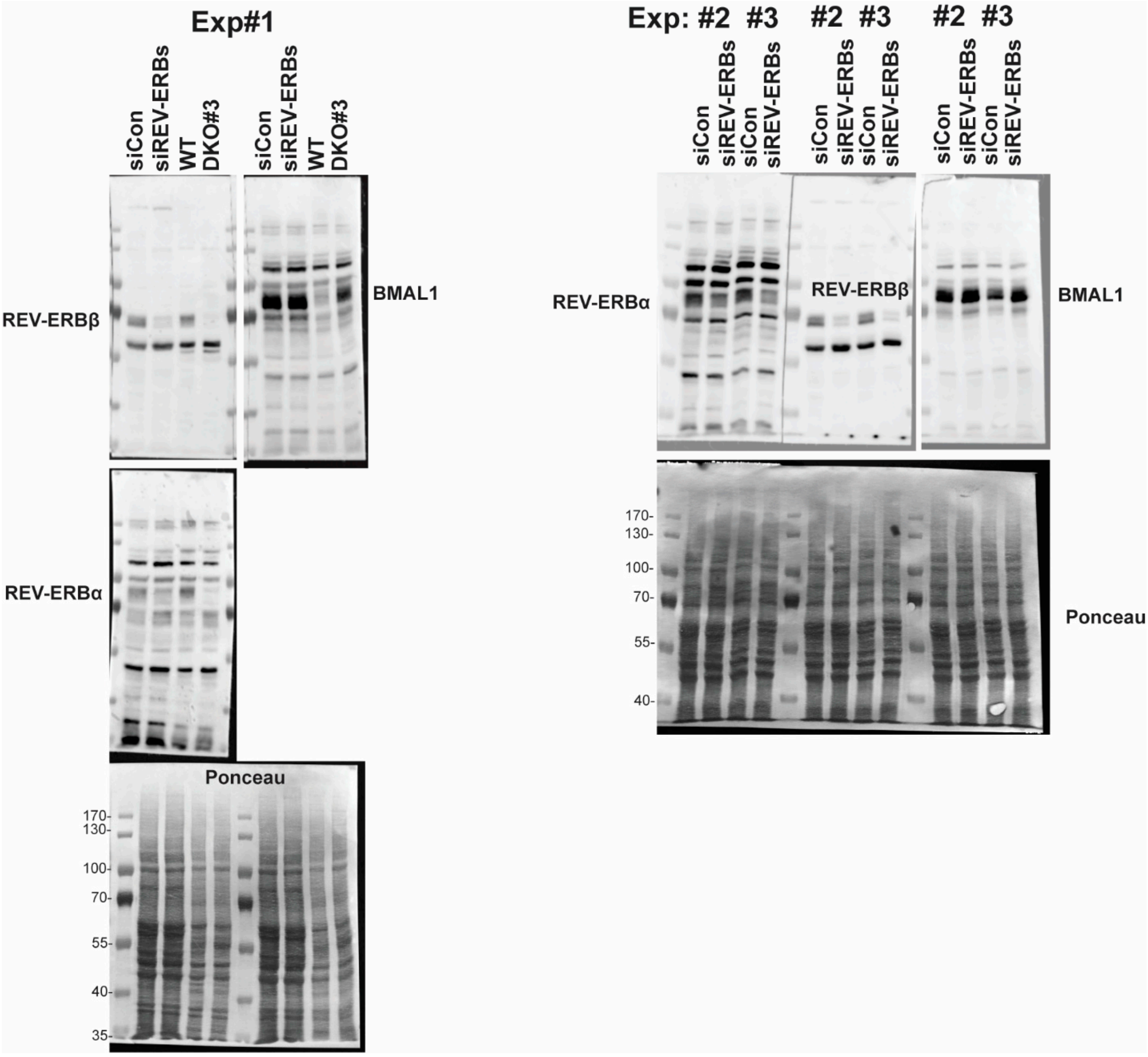


**Supplementary Figure S11. Original western blots and experimental replicates for Figure 4A.** N-TERT keratinocytes were transfected twice with 40 nM of either Control siRNA (siCon) or 20 nM of each of siRNAs targeting REV-ERBα and REV-ERBβ. Cell lysates were prepared 24 hr after the second transfection and examined by Western blotting for the indicated proteins. The transfection experiment was carried out on three biological replicates of cells at different passage numbers. In the first experiment, lysates from wild-type (WT) and REV-ERBα/β-double knockout (DKO) were used as a control.

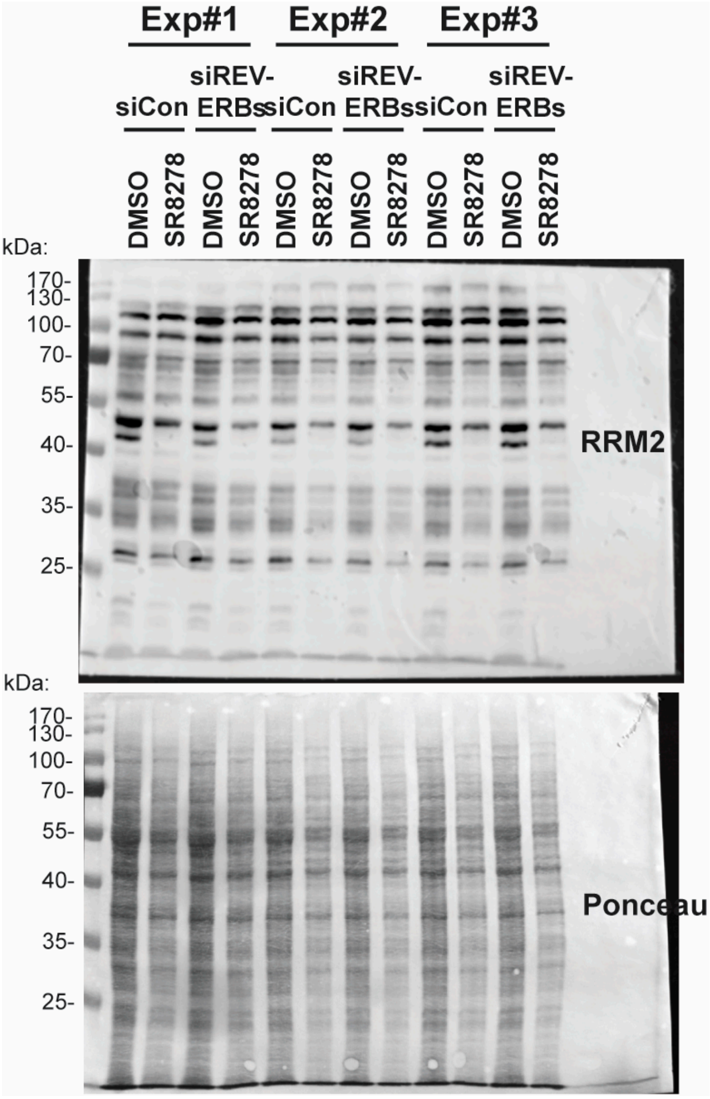


**Supplementary Figure S12. Original western blots and experimental replicates for Figure 4D.** N-TERT keratinocytes were transfected twice with 40 nM of either Control siRNA (siCon) or 20 nM of each of siRNAs targeting REV-ERBα and REV-ERBβ. Cells were then treated with 0.1% DMSO or 25 μM SR8278 for 48 hr. Lysates were then examined by Western blotting. The results of three independent biological replicate experiments are shown.
